# Benefit of BP Measurement in Pediatric ED Patients

**DOI:** 10.5402/2012/627354

**Published:** 2012-06-18

**Authors:** Karen M. Poor, Tamara Bostrack Ducklow

**Affiliations:** ^1^ED/ICU/Telemetry, HealthEast Woodwinds Health Campus, Woodbury, MN 55125, USA; ^2^Emergency Department, HealthEast St. John's Hospital, Maplewood, MN 55109, USA

## Abstract

*Introduction*. Obtaining blood pressures in pediatric emergency department patients is the standard of care; however, there is little evidence to support its utility. This prospective study assesses the benefit of BP acquisition in patients ≤5 years. *Methods*. Data were collected by the ED triage nurses on 649 patients in two community hospital EDs. Relationships between abnormal blood pressures and the patients' age, acuity, and calm versus not-calm emotional state were analyzed. *Results*. There were significant differences in the rate of elevated BPs in the calm and not-calm groups of patients. Overall, one- and two-year-old patients were more likely to have elevated BPs than those in other age groups. Very few patients in the sample had hypotension (1%). There was no relationship between Emergency Severity Index (ESI) acuity level and an abnormal BP. Nineteen percent of calm patients had elevated BPs, with 3.6% of patients in the stage two class of hypertension. *Conclusions*. There is limited benefit in obtaining BPs in children age of five or less regardless of whether the child is calm or not in ESI acuity levels 3 and 4.

## 1. Introduction 

The standard of care in emergency departments is to obtain a blood pressure (BP) during the triage of an injured or ill pediatric patients. Obtaining a BP in pediatric patients, particularly in children under the age of 5 years, is often difficult. Moro-Sutherland [1] notes that “an accurate BP measurement can be difficult to obtain in a young, active, or crying child; can be time-consuming; and requires the use of a properly sized cuff.” This process can be a discomforting or even traumatizing experience. Studies have shown that BP acquisition in the pediatric emergency population is inconsistent and variable [2–4]. 

BPs are obtained in the ED to assess for either hypotension or hypertension. Hypotension in children is considered to be a late sign of decreased tissue perfusion. Tachycardia, cool and pale distal extremities, prolonged capillary refill, weak pulses, depressed mental status, tachypnea, and decreased urinary output are present prior to a decrease in the systolic BP [5]. The incidence of hypertension in the pediatric population is thought to be approximately 3.6% [6], although, with the epidemic of obesity in children, that number may be higher [7]. Numerous studies have shown a correlation between hypertension in adolescence and cardiovascular disease later in life; however, that correlation does not seem to be strong until the patient is more than five years of age [8]. The Fourth Report on the Diagnosis, Evaluation, and Treatment of High Blood Pressure in Children and Adolescents advocates routine screening of the BP in patients over three years in a medical setting [7]. There is controversy over whether the ED is the setting in which to perform this screen [9]. 

The goal of this prospective study was to determine the benefit of BP measurement in pediatric patients less than six years of age in relation to their age, acuity, and emotional state. Our study hypotheses were that

1. the BP of a patient ≤5 years is more likely to be in the normal BP range with lower acuity;
2. the BP of a patient ≤5 years is more likely to be elevated when the patient is not calm;
3. the BP of a patient ≤5 years is more likely to be elevated in younger children.

## 2. Methods

This prospective observational study was done at Woodwinds Health Campus and St. John's Hospital EDs in the St. Paul, Minnesota metropolitan area. The two EDs see a combined 60,000 patients per year with 15,000 pediatric patients. The nonexperimental study was approved by the HealthEast System Institutional Review Board with a waiver of informed consent because vital sign acquisition is standard practice. Patients between the ages of six months and five years were included in the data collection as this is the most difficult group in which to obtain BPs. Data was collected on a convenience sample of patients between 0700 and 2330, seven days per week. Patients were excluded from the study if the patient had

- a previous diagnosis of hypertension;
- renal dysfunction, including renal failure, significant renal malformation, and glomerulonephritis;
- diabetes;
- significant congenital heart disease;
- chemical treatment with chemotherapy, immunosuppressant medications, or long-term corticosteroids.

Seventy-five ED triage nurses were educated on the study protocol, correct size of BP cuff, calming techniques, and study definition of calm versus not-calm emotional states. Reliability testing was not done because BP acquisition is a standard competency. To work in a triage area, the nurse has at least one year of emergency nursing experience, has passed a triage course, and participates in triage review education yearly.

Calming techniques included distraction and parental soothing as deemed appropriate for age, acuity, and behavioral state. Patients were determined to be “calm” if they were cooperative, still, and accepting of BP procedure. Patients were considered to be “not calm” if they were crying, fighting, or moving during the measurement. Each patient presenting to the ED was registered and then evaluated by the triage nurse. The blood pressure was obtained using a GE Procare 300 automated vital sign machine. Machines are calibrated yearly by the biomedical staff at each site to ensure accurate readings. The triage nurse chooses a blood pressure cuff that is one-third the size of the patients' arm. 

After the evaluation, the triage nurse assigned an acuity by using the Emergency Severity Index (ESI), an international standard for emergency department acuity designation [10]. The ESI acuity ranges from one to five, with one indicating a need for life-saving intervention and five indicating that the patient does not require any resources, such as procedures, laboratory, or radiology tests. After the patient was triaged, the triage nurse documented the all study data on a spreadsheet.

Blood pressures were analyzed using the Fourth Report's table “Blood Pressure Levels for Boys/Girls by Age and Height Percentile” [7]. Patients were considered to be in stage I hypertension if they were in the 95th–99th percentile plus five mm Hg and in stage II hypertension if they were in the 99th percentile plus 5 mm Hg. The patients were considered to be hypotensive if their systolic blood pressure fell below the Pediatric Advanced Life Support criteria of 70 + 2 multiplied by their age in years [5].  *P*  value and confidence interval calculations, as well as statistical analysis, were accomplished using SPSS version 15.0.

## 3. Results

Data on 680 patients who were ≤5 years were collected for this study. Of these, 21 patients were excluded because of missing data. This left 659 patients for inclusion in this study with an average age of 2.38 years. Fifty-seven percent of children had a normal systolic BP, where 42% of children had a normal diastolic BP. Roughly 6% of all patients had a lower than normal systolic BP, but only 1% had hypotension as defined by PALS criteria [5].

There was no correlation between hypotension and ESI acuity level. Six of the 7 patients with hypotension were classified as ESI level 4; one patient was an ESI level 5. There was no significant difference between the acuity levels and the incidence of elevated BP.

There were significant differences in the blood pressure when considering the state of calmness (Table 1). Unsurprisingly, nurses were able to obtain blood pressure readings more often in calm patients (83%) than not-calm patients (45%). Calm patients had a lower average systolic blood pressure (102 versus 111) and diastolic blood pressure (66 versus 76) compared to patients that were not calm (*P* < 0.001). Patients that were not calm were more likely to have a high systolic and diastolic pressure than patients who were calm (*P* < 0.001).

Because the number of patients with an ESI of 2 or 5 was very small, testing for differences between calm and not-calm groups was not done. The analysis of data from patients in ESI categories 3 and 4 is summarized in Table 2. Significantly more calm patients in the ESI 3 level had a BP obtained than not-calm patients (99% versus 57%). Patients who were not calm more often had high systolic BP (42% versus 12%) and diastolic BP (76% versus 41%). Of the calm patients in the ESI 3 group, 10% exhibited BPs that fell into the stage 1 hypertension category and 2% were in the stage 2 hypertension category. This contrasted with the not-calm patients who were 7% in stage 1 and 35% in stage 2.

Similar results were found for ESI 4 patients. A BP was obtained in significantly more calm patients than not-calm patients (97% versus 38%). Patients who were not calm more often had high systolic BP (59% versus 21%) and diastolic BP (77% versus 46%) compared to patients that were calm. Fifteen percent of calm patients in the ESI 4 category were in stage 1, with 6% in stage 2 as opposed to 33% of not-calm patients in stage 1 and 26% in stage 2.

The data revealed that patients who were calm were on average older (2.7 years of age) than patients that were not calm (1.5 years of age). The data (Table 3) showed a significantly higher number of one- and two-year-old patients who had systolic hypertension in the not calm group versus the calm group (*P* < 0.001). In total, 19% of all patients in the calm sample had BPs that were above the norm for BP in their age group. This is compared to 53% of all patients in the not-calm sample who had elevated BPs. The data showed that only a small percentage of patients (3.6% of calm and 3.3% of not calm) had BPs that were in the stage two class of hypertension. 

## 4. Discussion

The data did not support the hypothesis that patients with a lower acuity would have fewer abnormal BP readings. This may have been due to the small number of patients in the higher and lower levels of acuity. 

Results of this study showed that 24% of all patients had elevated BPs. This result is consistent with three other studies related to BP in children in the ED. Stewart et al. [11] collected BP data on 549 nonurgent patients ≥3 years; 26% were found to have elevated BPs at triage. Only 3.8–7.5% were found to have true hypertension on a follow-up BP check. The authors concluded that the utility of obtaining BPs on pediatric patients in nonurgent conditions was very low. Gilhotra and Willis [2] prospectively investigated both the frequency of BP measurement and the followup of abnormal BP values. They found that only 22.6% of children had an initial BP taken; of those, 30.7% of patients met criteria for hypertension. Approximately half of patients with high BP readings had their BPs remeasured; of those, only 1 in 10 children with abnormal consecutive elevated readings had a follow-up appointment after the ED visit. Silverman et al. [4] retrospectively reviewed 437 charts on patients aged 1 month to 18 years. They found that only 38% of abnormal BPs were repeated; of those, 55% were elevated. This study showed that the percentage of elevated BP in patients dramatically increased in not-calm states.

There are several plausible reasons for such a high rate of elevated BPs. Children in the emergency setting often present with problems inducing anxiety, fear, and pain [12], which triggers a sympathetic nervous system response. This includes tachycardia, tachypnea, and hypertension through a neurohormonal cascade. Children may also be prone to white coat hypertension [13], in which there is documentation of normal BP in nonhealth care settings with hypertension in the health care setting [14]. In both of these situations, an elevated BP is not indicative of true hypertension. Duncan et al. found that children 1–3 years of age were more likely to have elevated BPs during noncalm states [15]. This may be due to anxiety and fear causing muscular tension and/or fist clenching. 

The data in this study showed that the BP in the calm child was more likely to be in the normal range; however, 19% of calm patients had an elevated systolic BP and 44% of calm patients had an elevated diastolic BP. Multiple factors influence the accuracy and reliability of a blood pressure in children. First, the recommended method of BP acquisition in the child is through auscultatory means rather than the oscillometric method that is generally used in EDs. Second, children over 3 years should be in a seated position with feet on the floor after having a five minute quiet period, should have not ingested any stimulant drugs or food, and have the arm raised to the level of the heart [7]. Third, there is evidence that questions the accuracy of clinic measurement of BP [14] and known variability in vital sign accuracy. Edmonds et al. [16] conducted a study in which they found significant interrater variability between the two observers when obtaining vital signs on 140 patients. Podoll et al. found that 74% of the 390 children had BPs higher at the previsit vital sign station than when retaken in the examination room [17]. Because of these confounders to a definitive diagnosis of hypertension based on one BP reading, multiple BP measurements over multiple days or the use of ambulatory BP monitoring are recommended [7]. 

Finally, we reviewed recommendations from national groups related to acquisition of BP in pediatric emergency patients. The ESI Implementation Handbook discusses vital sign acquisition in all emergency patients [10]. Only ESI acuity 3 mandates vital signs, which are defined as heart rate, respiratory rate, oxygen saturation (when pertinent), and temperature on children less than 3. A policy statement on care of children in the ED, as accepted by multiple pediatric and emergency national groups, recommends that BP measuring and monitoring capability are available but views a full set of vital signs for children as the temperature, heart rate, and respiratory rate [18]. 

Although it is a standard of practice to obtain a BP in pediatric emergency patients, we found that this practice is not based on recommendations from national groups or on evidence from research.

## 5. Limitations

There are several limitations to this study. There may have been variability in the method of BP acquisition and the perception of whether the child was calm or not calm. The patient was not followed to obtain subsequent BP measures to assess for continued hypertension or followup by the ED provider. This study took place in two suburban communities and may not be generalized to populations with a higher level of acuity or hypertension.

## 6. Implications for Emergency Nurses

Nurses need to be aware that a child who is not calm will tend to have abnormally elevated BPs. Nurses should also be knowledgeable of children who should have BP acquisition done regardless of age or acuity, such as those with previous diagnosis of hypertension, renal dysfunction, including renal failure, significant renal malformation, glomerulonephritis, diabetes, significant congenital heart disease, or chemical treatment with chemotherapy, immunosuppressant medications, or long-term corticosteroids. Blood pressure acquisition in children ≤5 years has limited benefit in ESI categories three or four.

## 7. Conclusions

The standard in the ED community is to obtain BP in all patients, regardless of acuity or age. We found that, in ESI acuity levels 3 and 4, there is limited benefit in obtaining BPs in children age of five or less regardless of whether the child is calm or not. Pain, anxiety, and fear are predominant in this population on entry to the emergency setting. An unfamiliar and uncomfortable procedure such as BP acquisition leads to not only increased anxiety and fear but to potentially inaccurate diagnostic results.

## Figures and Tables

**Table 1:** Calm versus not-calm patients.

	Calm	Not calm	*P* value
BP obtained	471 (83%)	79 (45%)	<0.001
Unable to obtain BP	11 (17%)	98 (55%)	

SBP			

Average SBP	102	111	<0.001
Low	38 (8%)	5 (6%)	<0.001
Normal	343 (73%)	32 (41%)	
High	90 (19%)	42 (53%)	

DBP			

Average DBP	66	76	<0.001
Low	3 (1%)	1 (1%)	<0.001
Normal	263 (56%)	17 (22%)	
High	205 (44%)	61 (77%)	

**Table 2:** Blood pressures in ESI categories 3 and 4.

	ESI 3			ESI 4		

	Calm	Not calm	*P* value	Calm	Not calm	*P* value
	Number (%)	Number (%)		Number (%)	Number (%)	
BP obtained	127 (99%)	29 (57%)	<0.001	302 (97%)	43 (38%)	<0.001
Unable to obtain BP	1 (1%)	22 (43%)		8 (3%)	70 (62%)	

SBP category						
Low	10 (8%)	2 (7%)	<0.001	23 (8%)	3 (7%)	<0.001
Normal	101 (80%)	15 (52%)		214 (71%)	15 (35%)	
Stage 1 HTN	13 (10%)	2 (7%)		46 (15%)	14 (33%)	
Stage 2 HTN	3 (2%)	10 (35%)		19 (6%)	11 (26%)	

DBP category						
Normal	75 (59%)	7 (24%)	<0.001	163 (54%)	9 (21%)	<0.001
Stage 1 HTN	38 (30%)	10 (35%)		105 (35%)	11 (26%)	
Stage 2 HTN	14 (11%)	12 (41%)		32 (11%)	22 (51%)	

**Table 3:** Relationship between age and BP.

Age	6 mos–1 year			1 year			2 years			3 years			4 years			5 years		
	Calm	Not calm	*P*	Calm	Not calm	*P*	Calm	Not calm	*P*	Calm	Not calm	*P*	Calm	Not calm	*P*	Calm	Not calm	*P*
SBP																		
Normal	30 (67)	7 (54)	.52	54 (75)	13 (38)	<0.001	86 (87)	8 (44)	<0.001	67 (85)	4 (67)	.26	82 (80)	1 (50)	.38	62 (85)	4 (67)	.26
Hypertensive	15 (33)	6 (46)		18 (25)	21 (62)		13 (13)	10 (56)		12 (15)	2 (33)		21 (20)	1 (50)		11 (15)	2 (33)	

DBP																		
Normal	12 (27)	1 (8)	.26	18 (25)	6 (18)	.46	50 (51)	7 (39)	.45	57 (72)	2 (33)	.07	72 (71)	0 (0)	.09	56 (77)	2 (33)	.04
Hypertensive	33 (73)	12 (92)		54 (75)	28 (82)		49 (50)	11 (61)		22 (28)	4 (67)		30 (29)	100 (100)		17 (23)	4 (67)	

Numbers in parentheses are percentages.
